# Potential Therapeutic Role of Purinergic Receptors in Cardiovascular Disease Mediated by SARS-CoV-2

**DOI:** 10.1155/2020/8632048

**Published:** 2020-12-01

**Authors:** Fernanda dos Anjos, Júlia Leão Batista Simões, Charles Elias Assmann, Fabiano Barbosa Carvalho, Margarete Dulce Bagatini

**Affiliations:** ^1^Medical School, Federal University of Fronteira Sul, Chapecó, SC, Brazil; ^2^Department of Biochemistry and Molecular Biology, Federal University of Santa Maria, Santa Maria, RS, Brazil; ^3^Federal University of Health Sciences of Porto Alegre, Porto Alegre, RS, Brazil; ^4^Graduate Program in Biomedical Sciences, Federal University of Fronteira Sul, Chapecó, SC, Brazil

## Abstract

Novel coronavirus disease 2019 (COVID-19) causes pulmonary and cardiovascular disorders and has become a worldwide emergency. Myocardial injury can be caused by direct or indirect damage, particularly mediated by a cytokine storm, a disordered immune response that can cause myocarditis, abnormal coagulation, arrhythmia, acute coronary syndrome, and myocardial infarction. The present review focuses on the mechanisms of this viral infection, cardiac biomarkers, consequences, and the possible therapeutic role of purinergic and adenosinergic signalling systems. In particular, we focus on the interaction of the extracellular nucleotide adenosine triphosphate (ATP) with its receptors P2X1, P2X4, P2X7, P2Y1, and P2Y2 and of adenosine (Ado) with A2A and A3 receptors, as well as their roles in host immune responses. We suggest that receptors of purinergic signalling could be ideal candidates for pharmacological targeting to protect against myocardial injury caused by a cytokine storm in COVID-19, in order to reduce systemic inflammatory damage to cells and tissues, preventing the progression of the disease by modulating the immune response and improving patient quality of life.

## 1. Introduction

It took three months for a sudden outbreak of novel coronavirus disease 2019 (COVID-19) to become a pandemic by March 11, according to the World Health Organization (WHO) [1]. The new coronavirus causes severe damage to the respiratory tract (initially the upper and eventually the lower) sometimes resulting in acute respiratory syndrome. Studies showed that the COVID-19 pandemic emerged as a zoonosis, given the shared history of more than 40 patients in Wuhan, China, who were exposed at the Huanan Seafood Market [2]. By sequencing the entire viral RNA genome, it was revealed that this contamination may be due to human contact with certain types of bats, because SARS-CoV-2 (the causative agent of COVID-19) appears to be closely related to two coronaviruses responsible for acute respiratory syndrome, bat-SL-CoVZC45 and bat-SL-CoVZXC21 [3]. Researchers in Guangzhou, China, suggested that human contact with pangolins occurred, making these animals the likely biological source of the COVID-19 outbreak [4].

The inflammatory process mediated by SARS-CoV-2 is essential for the organism to effectively fight the invasion of the virus, because, after the initial recognition of the pathogens, immune cell recruitment then activates cascades in order to eliminate the pathogen and, finally, restore homeostasis to the injured tissue. Nevertheless, excessive and prolonged responses of cytokines and chemokines such as a cytokine storm (CS) can occur [5].

In particular, we wish to focus on immune responses involving the nucleotide adenosine triphosphate (ATP), an intracellular energy molecule released from various types of cells after damage, which accumulates at sites of tissue injury and inflammation as well [1]. After release, it can activate receptors or be rapidly decomposed by ectonucleotidases [2]. Extracellular ATP in low concentrations opens cation channels and sometimes leads to cell proliferation, while at high concentrations, it is a proinflammatory danger signal [3] that upregulates P2X purinoceptors located on immune cells (neutrophils, eosinophils, monocytes, macrophages, mast cells, and lymphocytes) [1, 4]. The ATP that is released due to the action of SARS-CoV-2 is an important mediator of inflammation, promoting the proliferation of immune cells and T cell activation [4], possibly contributing to the exacerbation of the immunological response and damaging the myocardium.

In the purinergic system, adenosine, a product of ATP decomposition, has a primary immunosuppressive action by inhibiting the proliferation of T cells and the release of proinflammatory cytokines. This occurs via hydrolysis of ATP in the extracellular medium through the action of ectonucleotidases, NTPDase converting ATP to adenosine 5′-diphosphate (ADP) and ADP to adenosine monophosphate (AMP). Consequently, ecto-5′-nucleotidase converts AMP to adenosine, which is ultimately degraded to inosine by the action of adenosine deaminase (ADA) [6]. This immune exacerbation leads to dysfunction of several organs, including the heart [5]. For these reasons, immunosuppressive substances that regulate inflammatory responses can increase the success rate of treatment and reduce the mortality rate in patients with COVID-19 [6].

## 2. COVID-19: The Current Pandemic Context

The first case of COVID-19 was reported in Wuhan, China, at the end of 2019. Within two months, it spread around the globe by virtue of its high level of contagion. Transmission can occur through direct contact or through respiratory droplets, as well as through contact with contaminated surfaces [7]. Most of the available case reports show mild to severe respiratory disease with fever, fatigue, cough, myalgia, and difficulty breathing [8]. The main sources of infection are patients with pneumonia infected by the new coronavirus; it is estimated that the infection has an average incubation period of 6.4 days and a basic reproduction number of 2.24–3.58 [3].

Coronaviruses are enveloped positive-chain RNA viruses belonging to the *Coronaviridae* family of the order *Nidovirales*. They are classified into four genera: *Alphacoronavirus*, *Betacoronavirus*, *Gammacoronavirus*, and *Deltacoronavirus* [2, 9]. Of these, only the gamma genus cannot infect mammals; however, due to their genetic organization, they are prone to mutations and possible host exchange because they have the largest genome among RNA viruses. The coronaviruses that have already been identified may just be “the tip of the iceberg,” with potentially newer and more serious zoonotic events to be revealed, because SARS-CoV and MERS-CoV are betacoronaviruses, as is the new SARS-CoV-2. SARS-CoV infected 8,000 people with a mortality rate of approximately 10%; MERS-CoV infected 1,700 people, with a mortality rate of 36% [2].

According to the Chinese Center for Disease Control and Prevention, which recorded approximately 44,500 confirmed cases of COVID-19, 81% had mild illness (asymptomatic or mild pneumonia); 14% were severe, including dyspnoea, hypoxia, or pulmonary involvement; and 5% became critically ill, characterized by respiratory failure, shock, or involvement in other systems. The overall case mortality rate was 2.3% without registration of noncritical cases [10, 11]. Patients with metabolic diseases and comorbidities, especially cardiovascular ones, may face greater risks of progressing to severe conditions associated with worse prognoses [10, 12, 13].

## 3. COVID-19 Stages and Progression

Siddiqi and Mehra [14] proposed a 3-stage classification model, recognizing that COVID-19 illness exhibited three grades of increasing severity, corresponding to distinct clinical findings, responses to therapy, and clinical outcome [15], COVID-19: mild, moderate, and severe. The severe classification describes patients with difficulty in breathing, or a respiratory rate (RR) greater than 29 breaths per minute at rest, or average oxygen saturation less than 92%, or partial pressure of arterial oxygen in the blood (PaO_2_/fraction of inspired oxygen concentration FiO_2_) less than or equal to 300 mmHg. Critically ill patients are characterized by respiratory failure requiring mechanical ventilation, shock, or combined failure of other organs [7].

The third stage of infection, characterized by extrapulmonary systemic hyperinflammation with elevated inflammatory marks, appears to be less common in COVID-19 patients [14]; however, these symptoms must be studied because the mortality rate is much higher in patients in the third and most severe stage. With higher prevalence in hospitalized patients in intensive care, 7.2%, 8.7%, and 16.7% of acute cardiac injury, shock, and arrhythmia, respectively, were observed in a clinical cohort of patients with COVID-19 [16].

Upon entering the cell, the virus uses four mechanisms to circumvent the immune response. Initially, it inhibits interferon type 1 (IFN-1), known as the initial alarm, characterized by the rapid expression [17]. Then, the virus inhibits STAT-1 [18] phosphorylation to interfere with IFN-1 signalling; the third defensive mechanism is the exaggerated and prolonged production of IFN-1 by plasmacytoid dendritic cells (pDCs) to cause exhaustion so that the influx of inflammatory macrophages/monocytes and activated neutrophils occurs, resulting in pulmonary immunopathology such as acute respiratory distress syndrome (ARDS) [19]. Finally, there is a CS that further weakens the immune system through IFN-1-mediated T cell apoptosis [5, 20].

It was also reported that the duration of symptoms in the severe group was longer than that in the mild group. It was also reported that the duration of symptoms was longer and the incidence of comorbidities was greater in the severe group. CD4+ and CD8+ T lymphocytes are reduced in the severe group, suggesting that they act as an important defence against SARS-CoV-2. Critically ill patients have other common characteristics that demonstrate the deterioration of the immune system, including atrophy of the spleen and lymph nodes, along with reduced lymphocytes in lymphoid organs, and much lower levels of lymphocytes, especially natural killer (NK) cells; the majority of infiltrated immune cells in lung lesions are monocytes and macrophages, with minimal lymphocyte infiltration [15].

According to reports, patients infected with SARS-CoV-2 may have symptoms similar to those of SARS 2002. In the first analysis, the initial symptoms were fever, dry cough, rhinorrhoea, headache, and fatigue, in addition to diarrhoea, odynophagia, anosmia, and ageusia. The scenario may worsen, although approximately 80% of patients have mild symptoms, with lymphopenia and interstitial pneumonia [21, 22]. When COVID-19 rapidly progresses, these immune and inflammatory responses are capable of creating a severe CS and releasing proinflammatory markers such as interleukin 2 (IL-2), interleukin 6 (IL-6), interleukin 7 (IL-7), interleukin 10 (IL-10), granulocyte colony-stimulating factor (G-CSF), gamma interferon-inducible protein 10 (IP-10), monocyte chemotactic protein-1 (MCP-1), macrophage inflammatory protein 1-alpha (MIP-1*α*), and tumour necrosis factor-alpha (TNF-*α*) [21, 23]. In this situation, an exaggerated immune system response and an excessive inflammatory response may cause multiple organ failure and ARDS and may possibly cause death [21, 22].

Burnstock (2017) claims that purinergic signalling plays a major role in both physiology and pathophysiology in the heart, brain, gut, and lung. However, in the pandemic context in which elderly patients are more susceptible to complications, it is necessary to consider the age-related changes of purine receptors and their relationship with the following: (a) diseases of the central nervous system such as epilepsy, brain injury, psychiatric disorders, and neuropathic pain; (b) cardiovascular diseases such as hypertension, atherosclerosis, thrombosis, and stroke; (c) diseases of the airways such as chronic obstructive pulmonary disease, airway infections, asthma, lung injury, pulmonary fibrosis, cystic fibrosis, lung tumours, and chronic cough; and (d) gut disorders such as ulcerative colitis, Crohn's disease, irritable bowel syndrome, diarrhoea, and constipation.

## 4. COVID-19 and Cardiovascular Disease

Cardiac manifestations of coronavirus infections may be understood as a combination of factors, and damage may be caused directly or indirectly by viral infection [24, 25]. A recent study reported that patients with previous SARS infections had cardiovascular consequences and myocardial injury [26, 27] with systolic and diastolic dysfunction followed by heart failure, arrhythmias, and death [13]. These findings suggest that the SARS coronavirus subfamily has the potential to infect and alter cardiac tissue, potentiating myocardial damage [26–28].

Lau et al. [29] reported palpitations in the form of tachycardia at rest or light effort in patients who were recovering from SARS. The possible causes include cardiac arrhythmia, anaemia, state of anxiety, impaired lung function, thyroid dysfunction, and autonomic dysfunction [29]. A meta-analysis suggested that patients with underlying cardiovascular diseases would be more likely to be infected with MERS-CoV [30], while other studies showed that, during periods of high influenza activity, there is a greater propensity for shock-treated ventricular arrhythmias, in the context of systemic, arterial, and severe myocardial inflammation [12, 31].

The primary diagnostic tests (Table 1) used to diagnose COVID-19 with cardiovascular involvement include N-terminal probrain natriuretic peptide (NT-proBNP). Higher levels are associated with high risk, worse prognoses, and intensive care unit (ICU) admission [23, 32, 33]. A high-sensitivity assay for troponin may be helpful for risk assessment in patients requiring ICU care and myocardial injury [23, 34]. Patients with higher levels of D-dimer may require ICU care [23, 35]. Procalcitonin (PCT) is used to assess the need for care in the ICU. Complete blood counts reveal leukopenia and lymphocytopenia [23, 32, 36] High levels of ferritin signal poor outcomes [23, 32, 36, 37]. High concentrations of IL-6 are associated with worse prognoses [32, 36, 38]. Cardiac computed tomography (CT) is used in uncertain cases with elevated troponins with or without obstructive coronary artery disease. In ECG, the most common changes include ST and PR segment elevation and T and Q waves. Echocardiography shows myocardial systolic dysfunction and demonstrates myocyte necrosis and mononuclear cell infiltration [23].

A cohort study of 416 patients associated cardiac injury with mortality in patients with COVID-19 at Renmin University Hospital in Wuhan, China. The average age was 64 years, and the majority were women. The most common symptoms were fever (80.3%), cough (34.6%), and shortness of breath (28.1%). Cardiac injury was present in 82 patients, and the associated comorbidities were hypertension (*p* < 0.001), elevated leukocyte counts (median: 9,400), and elevated levels of CRP (median: 10.2), PCT (median: 0.27), CK-MB (median: 3.2), myohaemoglobin (median: 128), hs-TnI (median: 0.19), NT-proBNP (median: 1,689), aspartate aminotransferase (AST, median: 40), and creatinine (median: 1.15). In addition, there was diffuse mottling and ground-glass opacities on radiographic findings (64.6%). Frequent complications in those with cardiac injury were ARDS (58.5%), acute kidney injury (8.5%), electrolyte disturbances (15.9%), hypoproteinaemia (13.4%), and coagulation disorders (7.3%). The mortality rate among patients with cardiac injury (51.2%) was much higher than that among those without cardiac injury (4.5%) [39].

There was a case report of a patient with pneumonia and cardiac symptoms who demonstrated elevated troponin I (Trop I) (11.37 g/L), myoglobin (Myo) (390.97 ng/mL), and NT-proBNP (22,600 pg/mL). The ECG showed sinus tachycardia; echocardiography revealed an enlarged left ventricle (61 mm), diffuse myocardial dyskinesia, low left ventricular ejection fraction (LVEF) (32%), and pulmonary hypertension (44 mmHg) [40].

**Figure 1:**
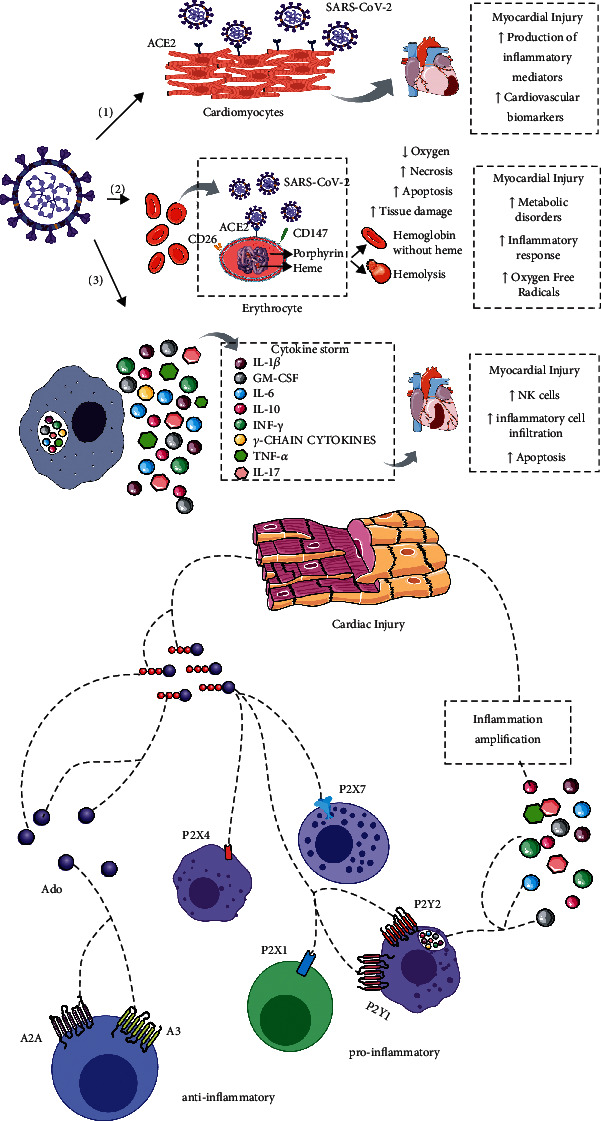
The cardiovascular system can be affected by COVID-19 and can cause myocardial injury. We focused on three mechanisms: (1) by the angiotensin-converting enzyme II (ACE2), (2) by the respiratory dysfunction and hypoxemia due to COVID-19, and (3) by the cytokine storm syndrome, which results in damage to the myocardium. Myocardial injury releases extracellular nucleotide adenosine triphosphate (ATP), a proinflammatory danger signal via the upregulation of P2 purinoceptors located on immune cells (neutrophils, eosinophils, monocytes, macrophages, mast cells, and lymphocytes). ATP released due to the action of SARS-CoV-2 is an important mediator of inflammation, promoting the proliferation of immune cells and T cell activation, possibly contributing to the exacerbation of immunological responses and myocardial damage.

Recent studies suggest that cardiac injury is due to the close relationship between the course of the disease and the cardiovascular system (see Figure 1). We focus on three main mechanisms resulting from COVID-19 responsible for myocardial injury: the angiotensin-converting enzyme II (ACE2), cytokine storm syndrome [13, 23, 41], and respiratory dysfunction and hypoxemia due to COVID-19, resulting in damage to the myocardium [42].

### 4.1. Myocardial Injury and ACE2

To better understand the myocardial injury caused by ACE2, it is necessary to remember the pathophysiology of COVID-19. The ACE2 protein is the cellular receptor through which SARS-CoV-2 enters cells [39, 40]; it is a membrane protein necessary for viral binding and internalization, similar to SARS-CoV [43]. After binding, activation of viral peak glycoprotein occurs and the C-terminal segment of ACE2 is cleaved by proteases such as TMPRSS2 and FURIN that are expressed in lung tissue [13, 26, 43].

Hair cells and goblet cells are concentrated in the upper nasal region and have high levels of ACE2 and TMPRSS [44], a fact that facilitates the entry of viruses in host cells, because serine protease assists with cleavage and glycoproteic activation of the viral envelope [45]. When infected, the respiratory epithelial cells undergo ciliary damage and become vacuolated [46]. In this situation, there is the production of inflammatory mediators that promote nasal secretions, inflammation, and local swelling, as well as stimulate sneezing, obstruction of the airways, and increase of the temperature of the mucosa [47]. In this manner, SARS-CoV-2 inserts into type 2 pneumocytes, cardiomyocytes, and macrophages via ACE2 [23]. In response, there may be myocardial, endothelial, and microvascular damage and dysfunction, plaque instability, and myocardial infarction (MI) [46].

In the heart, ACE2 is also highly expressed, and in some states of overactivation such as atherosclerosis, hypertension, and congestive heart failure, the effects of angiotensin II are neutralized [48, 49]. When a hospitalized patient has an early assessment, cardiac damage and clotting are monitored continuously; parameters such as cTnI, NT-proBNP, and D-dimer are elevated; and cardiac injuries can be identified, increasing the chance of predicting possible complications in COVID-19 [23]. Therefore, comorbidities that promote changes in metabolism can trigger a series of biochemical events that lead to increased expression of the *ACE2* gene, causing these patients to have an exacerbation of infected cells and therefore more severe clinical manifestations [49].

There is no relationship between the use of ACE inhibitors or angiotensin receptor blockers (ARBs) and the adverse outcomes [23]. ACE2 is widely distributed in the heart, testicles, kidneys, and lungs, where it functions as an antagonist of the classic RAS system; it is able to protect against organ damage in cases of hypertension, diabetes, cardiovascular diseases, and severe lung injuries, including ARDS, which is associated with high mortality [50]. Thus, ACE2 participates in cardiovascular homeostasis and is a functional receptor and gateway for coronavirus, as well as coronavirus 2. In this context, there is a connection between SARS-CoV-2 and ACE2 in that the negative regulation of this receptor is closely associated with cardiac protection [51].

### 4.2. Myocardial Injury and Hypoxia

Hypoxemia caused by COVID-19 can result in serious damage to myocardial cells [42]. A recent review pointed out that the supposed basic pathophysiological interaction between haemoglobin and SARS-CoV-2 is made by ACE2, CD147, and CD26 receptors located on erythrocytes or blood precursor cells. After viral endocytosis, there is a link between the cell receptor and the spike proteins that bind to the porphyrin that attacks haemoglobin in its heme portion. This would result in haemolysis or the formation of dysfunctional haemoglobin without heme with impaired transport of oxygen and carbon dioxide [52]. Another marker of haemolysis is the increase of lactate dehydrogenase (LDH) levels [53] that differentiates between the severe and mild cases of COVID-19, in addition to a reduction in haemoglobin levels [54].

Tissue damage such as necrosis and apoptosis is associated with loss of oxygen that causes the degeneration of mitochondria, giving rise to anaerobic glycolysis instead of the Krebs cycle and oxidative phosphorylation [52]. Cytotoxic cell death was also found to be caused by excessive autophagy [42]. Several metabolic disorders, inflammatory processes, oxygen free radicals, and several signalling pathways act to damage the myocardium [53].

### 4.3. Myocardial Injury and Cytokine Storm

Another possible mechanism of myocardial damage is CS [13, 27]. Laboratory data from patients with severe COVID-19 identified a marked inflammatory process, or CS, which is common in viral infections [55]. This hypercytokinaemia is characterized by elevation of levels of inflammatory markers associated with cytopenia and hyperferritinaemia [56]. Many studies [7, 46, 51, 56–62] of CS confirmed that levels of IL-2, IL-7, IL-10, TNF-*α*, granulocyte colony-stimulating factor (G-CSF), IP-10, MCP-1, and macrophage inflammatory protein 1-alpha (MIP-1*α*) were significantly higher in patients admitted to the ICU for COVID-19, although other studies [7] demonstrated little difference between IL-4, IL-17, and TNF and significant differences in IL-6 and IL-10 levels, suggesting a systemic inflammatory process in critically ill patients.

According to Ye et al. [5], the rapid replication of the virus induces the delayed release of IFN-*α*/*β*, as well as the influx of pathogenic inflammatory mononuclear macrophages that, upon receiving signals of activation through IFN-*α*/*β* receptors on their surface, attract monocytes, CCL2, CCL7, and CCL12. When activated, mononucleated macrophages produce TNF, IL-6, IL-1*β*, and inducible nitric oxide synthase proinflammatory cytokines [63]. Accordingly, another factor that contributes to the failure of viral clearance is the action of other proinflammatory cytokines derived from IFN-*α*/*β* or mononuclear macrophages responsible for inducing apoptosis of T cells. IFN-*α*/*β* and IFN-*γ* directly induce damage to lung tissue through ligand Fas-FasL inflammatory cell infiltration and cause apoptosis of cells in the pulmonary epithelium, causing vascular leakage, alveolar oedema, and hypoxia [5]. In short, this condition is due to the failure of the action of NK cells and cytotoxic T lymphocytes to eliminate activated macrophages, a condition called haemophagocytic histiocytosis, responsible for immune hyperactivation leading to an exacerbation in the production of proinflammatory cytokines and consequent injury [64]. These findings suggest that CS is closely related to the severity of the disease [65].

Reports revealed that preexisting cardiovascular conditions are risk factors, given that among the 99 patients hospitalized for pneumonia associated with SARS-CoV-2, 40% have such conditions [66]. At Zhongnan Hospital of Wuhan University, 26% of the 138 hospitalized patients required intensive cardiological therapy, of which 16.7% manifested arrhythmias and 7.2% developed acute coronary syndrome (ACS) [16].

## 5. Cardiac Consequences due to SARS-CoV-2

### 5.1. Myocarditis, Acute Coronary Syndrome, and COVID-19

Myocarditis is a specific inflammatory disease of the heart characterized by myocardial injury; viral infection is one of the most common causes [67]. Viral myocarditis has pathological phases and begins with virus-mediated myocyte lysis that generates an innate immune response through the release of proinflammatory cytokines. Myocyte lysis releases proteins that can be presented by antigen-presenting cells due to the similarity of epitopes to antigens such as the myosin heavy chain. After the acute phase, the immune system activates the immune response acquired through cellular activation of B and T lymphocytes that activate the inflammatory cascade, attracting macrophages. This inflammatory disease can be aggravated by the CS described above, including IL-6, and is explained by direct tissue injury and cytotoxic T lymphocytes that cause necrosis, local or generalized inflammation of the myocardium, and ultimately ventricular dysfunction [68].

Cardiovascular magnetic resonance imaging is used for the diagnosis of acute myocarditis [69]; it detects typical signs of acute myocardial injury. Recent studies have reported the use of this test during SARS-CoV-2 infection [70]. Endomyocardial biopsy (EMB), long considered the gold standard diagnostic test, directly demonstrates myocyte necrosis and mononuclear cell infiltration [71]. Clinical diagnosis can be made through clinical symptoms such as chest pain, as well as elevation of troponin T (TnT) or troponin I (TnI). The diagnosis of myocarditis by the ECG electrocardiogram is limited; nevertheless, the most common changes include ST segment elevation, T and Q waves, PR depression [67], myocardial ischaemia [39], and arrhythmias including ventricular tachycardia and ventricular fibrillation [69].

ACS is one of the most dangerous types of coronary heart disease (CHD). Patients with acute viral myocarditis commonly present with chest pain, elevated troponin levels, wall motion abnormalities, and ST segment depression or elevated T waves due to the development of ACS [72].

### 5.2. Arrhythmia and COVID-19

Viral infections promote electrolyte imbalances, metabolic dysfunction, nonmyocardial inflammatory processes, and activation of the central nervous system, all of which are factors that predispose to cardiac arrhythmia [23]. According to Kang et al. [73], the first symptom of COVID-19 may be arrhythmia, being an indicator of cardiac impairment of recent onset and progression. This electrolyte imbalance reduces the connection between the RAAS system and the SARS-CoV-2 viruses by increasing the degree of hypocalcaemia [74]. Arrhythmia is also associated with myocarditis. The main clinical manifestations are atrial and ventricular fibrillation, ventricular tachycardia, and conduction block. In addition, there are cases in which some patients present with cardiovascular and respiratory symptoms. In patients who were not admitted to the ICU, the rate of arrhythmia was 7%; by contrast, 44% of those who manifested arrhythmias were admitted [23]. Troponin levels appear to be associated with a greater number of malignant arrhythmias seen by patients with increased Tn levels, with higher mortality rates than those with lower levels of Tn [73]. A recent study pointed out that constant monitoring is necessary by means of the ECG of patients who already have inherited or acquired arrhythmias, and many protocols include medications that promote worsening of arrhythmogenic activity; for example, chloroquine and hydroxychloroquine can cause increased QT intervals [74].

### 5.3. Coagulopathy and COVID-19

Depending on the situation, SARS-CoV-2 activates or deactivates the complement system and induces vascular endothelial damage by increasing permeability and by the formation of inflammatory thrombi. In patients with COVID-19, thrombus formation must be activated by the fibrinolytic system which, when activated, releases fragments of fibrin degradation (D-dimers) in the circulation and overtraining of thrombin, a state of hypercoagulation in patients with infection [32, 38, 75]. Blood vessels are altered in two ways: type L, characterized by mild damage to the alveolar space, and type H, characterized by involvement of the alveolar space [38].

A recent study indicated four pathways responsible for the imbalance, contributing directly to the formation of thrombi. The first pathway involves a CS syndrome that causes the release of proinflammatory and hyperactive cytokines from the immune system such as IL-1*β* and IL-6 already mentioned in this study. This exacerbation of the active immune response is an extrinsic cascade of coagulation caused by stimulating tissue factor expression in cells of the immune system. In the second pathway, suppression of the fibrinolytic system occurs, or there is a reduction by the activity of the urokinase-type plasminogen activator or by increases in the inhibitor responsible for activating plasminogen. A third pathway involves the action of proinflammatory cytokines on plaques that cause them to activate and bind to the endothelium, causing damage. The fourth and final route involves direct damage caused by the inflammatory process and immune responses to endothelial damage with the contribution to the formation of thrombi [76].

Tang et al. [38] stated that hypoxia due to COVID-19 is possibly related to the formation of thrombi by increasing blood viscosity and by the signalling pathway dependent on the hypoxia-inducible transcription factor, because the dissection of lungs of critically ill patients revealed microthrombosis in small vessels [75]. In addition, there are a number of factors that increase the risk of thrombosis and pulmonary embolism, including obesity, advanced age, and immobilization in hospital beds [77]. Virchow's triad explains the increased risk of venous thromboembolic disease in critically ill patients admitted to the ICU, as there are venous stasis due to prolonged bed rest, prothrombotic changes due to the exacerbated action of the immune system, and endothelial injury. These patients are candidates for thromboprophylaxis, and the following parameters should be monitored: D-dimer, prothrombin time, platelet count, and fibrinogen, in order to control and improve prognoses [78].

### 5.4. Myocardial Infarction and COVID-19

Patients with comorbid cardiac impairments are likely to suffer complications caused by COVID-19 [7, 15, 21, 23, 38], especially acute events such as ischaemia due to the formation of clots developed as a result of systemic responses to viral invasion. According to Guzik et al. [23], the pathophysiology of CS that contributes to endothelial dysfunction and high expression levels of ECA2 in cardiac tissue may be localized microvascular inflammation that causes severe myocardial infarction. A study found that acute myocardial ischaemia was present in two of five patients who died from SARS and MERS, with MI type 2 being the most common subtype of virus infections [23].

## 6. Potential Therapeutic Manipulation of the Purinergic System

The purinergic system is characterized by the action of extracellular nucleotides and nucleosides that are degraded by the action of several ectonucleotidases [79]. The hydrolysis of ATP to adenosine (Ado) starts with the action of ectonucleotidases, NTPDase in adenosine 5′-diphosphate (ADP) and ADP in adenosine monophosphate (AMP). Consequently, ecto-5′-nucleotidase converts AMP to adenosine which is ultimately degraded to inosine by the action of adenosine deaminase (ADA) [6]. ATP is an intracellular energy molecule, but it can be released from various types of cells after damage. Thus, after release, it can activate receptors or be rapidly decomposed by ectonucleotidases [79].

Purinergic signalling receptors are classified into two main groups: P1 nucleoside receptor and P2 purinoceptor. The first group, P1 adenosine receptors, has four subtypes (A1, A2A, A2B, and A3). The second group, P2, can be classified into two subfamilies: P2X ionotropic nucleotide receptors, which are ligand-gated ion channels selective for cations with seven subtypes (P2X1-7), and P2Y metabotropic nucleotide receptors, which are G protein-coupled receptors with eight subtypes (P2Y1-2, P2Y4, P2Y6, and P2Y11-14) [76].

According to Tang et al. [75], in the immune system, purinergic components perform important regulatory functions, including mitogenesis and DNA synthesis in the cells of the thymus, macrophage activation and death, aggregation of neutrophils, secretory responses in basophils, chemotactic responses in eosinophils, and release of proinflammatory factors. The system also modulates proliferative responses in lymphocytes, release of histamine, and degranulation of mast cells and mediates intercellular Ca^2+^ waves in mast cells. Thus, the expression of receptors and ectonucleotidases in immune cells varies according to the amount of nucleotide and nucleoside available in the extracellular medium in normal conditions or in the context of certain diseases [79].

Immune system cells express receptors for adenosine. They have specific names, as follows: adenosine A1 receptor (ADORA1), adenosine A2A receptor (ADORA2a), adenosine A2B receptor (ADORA2b), and adenosine A3 receptor (ADORA3). The adenosine receptors ADORA2a and ADORA2b are associated with Gs protein inducing cAMP production while ADORA1 and ADORA3 with Gi protein which inhibits cAMP production [80].

Studies show anti-inflammatory functions of A2A signalling as stop signals of activation of different immune-inflammatory effector cells [81]. They are expressed in lymphocytes and neutrophils and are involved in the development of regulatory T cells (Treg) that express CD39 and CD73 and thus negatively regulate the activation of T cells. A2A also inhibits macrophage adhesion, macrophage recruitment, and transition of M1 to M2 by proinflammatory cytokines acting as stop signals [81]. These receptors suppress inflammation in a delayed, negative feedback manner because of inhibition by cAMP of molecular intermediates of proinflammatory signalling pathways, thereby allowing for the “acquisition” of an immunosuppressive “OFF button” and creation of a time window for immunomodulation [82]. Furthermore, adenosine plays an important anti-inflammatory role through T cell regulation, proliferation, activation, and cell death [77]. A pioneering study of Ado receptors evaluated the effects of reducing pH from 7.4 to 6.8; the authors concluded that the A2A adenosine receptor mediated the dilation of the mesenteric arterial bed of the rats and that the response to activation of this receptor was potentiated by a reduction in pH that was similar to that observed in ischaemic conditions [83]. In addition, signalling by A3 inhibits neutrophil degranulation in neutrophil-mediated tissue injury, TNF-*α* and platelet activation, and factor-induced chemotaxis of human eosinophils [78].

Adenosinergic inhibition of synaptic potentials was significantly enhanced in hippocampal slices from aged rats, contributing to age-related decline in synaptic efficacy. A1 and A2A receptor binding was modified in the aged striatum, hippocampus, and cortex of the rats. Another change observed in these animals was reduced adenosine A1 receptor and G*α* protein coupling in the ventricular myocardium during ageing [84]. These findings suggest that changes of purines should be considered relevant contributors to the more serious consequences of COVID-19 in elderly patients [85].

A recent study hypothesized that a process called viral sepsis is crucial to the disease mechanism of COVID-19, like that of severe influenza infection. The cytokine storm might play an important role in immunopathology in severe or critical cases resulting in uncontrolled inflammation [86]. There is interest in modulating A2A receptors to control sepsis. In vivo studies were provided, testing the prediction that the absence would lead to increased inflammation and increased tissue damage using mice deficient in the A2A receptor gene. Agonists did indeed prevent tissue damage models in the heart, kidney, lung, skin, vascular smooth muscle, and spinal cord [82].

From another point of view, despite the fact that ATP is an important intracellular molecule, it accumulates at sites of tissue injury and inflammation as well [87]. Extracellular ATP in low concentrations opens cation channels and sometimes leads to cell proliferation, while in high concentrations, it is a proinflammatory danger signal [88] that upregulates P2X purinoceptors located on immune cells (neutrophils, eosinophils, monocytes, macrophages, mast cells, and lymphocytes) [87, 89]. The ATP that is released due to the action of SARS-CoV-2 is an important mediator of inflammation, promoting the proliferation of immune cells and T cell activation [89], possibly contributing to the exacerbation of the immunological response and damaging the myocardium.

Regarding subtypes of ATP receptors (P2X1-7), ligand-gated ion channels [90] P2X1, P2X4, and P2X7 receptors play central roles in inflammation because they are expressed on T and B lymphocytes and NK cells [91]. Therefore, it is possible to infer a close relationship in the expression of these receptors in myocarditis, a consequence of myocardial injury during acquired immune responses [68].

The activation of T lymphocytes is favoured by the P2X1 receptor (P2X1R), which allows the entry of calcium and the activation of the transcription factor NFAT [92], while the P2X4 receptor (P2X4R) is associated with an early inflammatory mediator [91]. In addition, P2X4R is expressed at high levels of mRNA in immune cells and is the main receptor responsible for the entry of calcium into the cell. Another mechanism involves extracellular calcium levels dramatically reducing; channels are opened, allowing larger molecules to enter and favouring apoptosis [92]. In relation to pH, high ATP concentration in the lysosomes does not activate P2X4 at low concentration. The study showed that P2X4 is functioning as a Ca^2+^ channel after the fusion of late endosomes and lysosomes; P2X4 becomes activated by intralysosomal ATP only in its fully dissociated tetra-anionic form, when the pH increases to 7.4 [93]. P2X4R can act as an initial immune response, while the P2X7 receiver (P2X7R) amplifies inflammatory signals [91].

According to Antonioli et al., during infection, the surface expression of P2X4 receptors reduced and decreased ATP-evoked currents without altering total P2X4 receptor protein levels. Another point of the study was that inflammatory stimuli elicit rapid trafficking of P2X4 receptors to the macrophage cell surface, causing increased Ca^2+^ influx, thereby promoting their activity. After termination of macrophage activation, a feedback mechanism develops to curb P2X4 receptor trafficking to the cell membrane and function, probably aimed at facilitating the resolution of inflammation [94]. Another study showed that expression levels of P2X4 in myeloid cells were higher in males than in females, with potentially important consequences for several pathologies; eosinophils were by far the cell type expressing the highest level of P2X4 on the cell surface, suggesting that ATP-dependent activation could be important in the eosinophil biology in the context of COVID-19 [95].

P2X7R can be responsible for CS by releasing inflammatory cytokines and chemokines IL-1, IL-2, IL-6, IL-18, IL-1*β*, and IL-1*α* [91]. This suggests that P2X7R blockers may be beneficial for COVID-19 patients with exacerbated immune responses such as myocarditis, abnormal coagulation, arrhythmia, acute coronary syndrome, and myocardial infarction, as described above [38, 41, 65, 67, 73].

Extracellular ATP can increase immune responses, CS, and myocardial injury by the action of macrophages that express elevated levels of P2Y1R and P2Y2R and are important immune cells for production of inflammatory mediators such as TNF-*α*, IL-1*β*, and IL-6, as well as amplification of immune responses by NO [96]. The P2Y2R receptor has also been described as a regulator of mucus production on airway epithelia. ATP may reach concentrations capable of promoting P2Y2 receptor activation and promotes mucin secretion via complex Ca^2+^ and diacylglycerol- (DAG-) regulated mechanisms. Therefore, they believed that the P2Y2 receptor has promising perspectives as a therapeutic target to promote the otherwise poor ASL volume production associated with the pathophysiology of inflammatory airway diseases [97].

**Figure 2:**
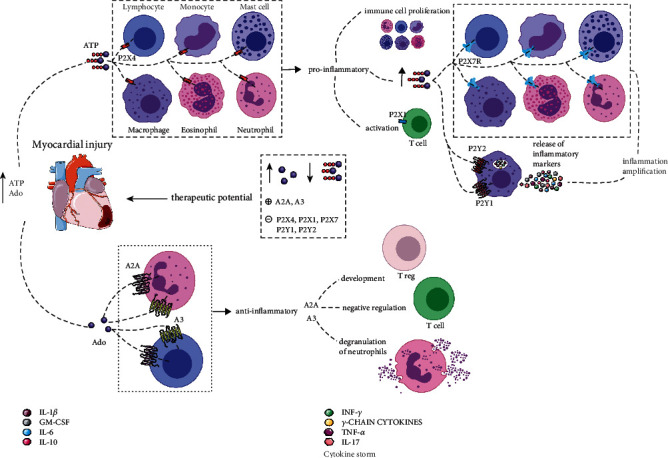
Myocardial injury increases levels of ATP and Ado such that different purinergic receptors are signalled. Initially, ATP signals P2X4 located on the membrane of immune cells such as mast cells, macrophages, and neutrophils. In response, there is the promotion of the inflammatory condition and the increase in ATP, promoting the proliferation of these immune cells, activating T cells by the P2X1 receptor, and signalling the P2X7 receptors of immune cells and the P2Y1 and P2Y2 in macrophages that trigger the release of inflammatory markers such as IL-1*β*, GM-CSF, IL-6, IL-10, INF-*γ*, *γ*-chain cytokines, TNF-*α*, and IL-17, amplifying inflammation. When increases in Ado, A2A and A3, are signalled, the first is responsible for the development of Tregs cells and for the negative regulation of T cells, while A3 is responsible for the degranulation of neutrophils. Thus, as a therapeutic potential for improving myocardial injury, deeper reductions in Ado and ATP occur, as well as positive modulation of A2A and A3 and negative modulation of P2X4, P2X1, P2X7, P2Y1, and P2Y2.

Regarding therapeutic potential, ATP is also a viable candidate. A study showed that removing extracellular ATP in a model of LPS-induced systemic inflammation in mice through the action of the enzyme that degrades ATPase protected against mortality associated with a significant reduction of proinflammatory cytokines that induce cell death (TNF, IL-1, and anti-inflammatory cytokine IL-10) [98]. Another study showed that ATP hydrolysis to adenosine (an anti-inflammatory mediator) by ectonucleotidases CD39 and CD73 suppressed the production of proinflammatory cytokines [91] (see Figure 2).

### 6.1. Purinergic Signalling in Ischemia-Reperfusion and Hypoxia

In myocardial ischaemia, positive modulation of the P2Y2 subtype looks promising, because it was shown to be protective during ischaemia-reperfusion in mouse hearts; however, in another study, they were able to increase cell death during hypoxia while P2Y4 acted as protectors in cultured cardiomyocytes [9]. In addition, the ischaemia-reperfusion picture reduced gene expression and protein content of purinergic receptors of the P2Y2 subtype and increased gene expression and protein content of the P2X7 subtype. Thus, treatment with the agonist of the P2Y2 subtype 2-thio-UTP and with the antagonist of the P2X7 subtype Brilliant Blue improved myocardial function parameters, reduced cell death, and increased myocardial expression of antiapoptotic markers after ischaemia-reperfusion [99].

In hypoxic conditions, with hypoxia-induced factor 1-alpha being the main response factor, the production of extracellular CD73-derived adenosine (ecto-5′-nucleotidase/NT5E) is considered an important pathway in the attenuation of induced inflammation [81, 99, 100] and protection of several central bodies [101–103]. Once in these conditions, Ado activates protein kinase C and improves mitochondrial function, modulating mitochondrial-sensitive potassium channels [104], ratifying the notion proposed by Burnstock and Pelleg, in which nucleotides are contributors to the hypoxia, while Ado is generally protective [105].

ATP exerts varying effects on vascular tone, acting as a constrictor or dilator, depending on the situation, such that, in endothelial cells through P2 receptors, there is the release of relaxing factors derived from the endothelium that diffuse to the vascular smooth muscle, inducing vasodilation. However, in pathophysiological situations such as hypoxia and ischaemia, the main source of intraluminal ATP is likely to be endothelial cells in sufficient quantities for the activation of local P2R [106].

Released with norepinephrine as a cotransmitter by sympathetic nerves, ATP acts as a vasoconstrictor by binding with P2X receptors located in vascular smooth muscle, whereas P2Y receptors, located in the vascular endothelium, mediate vascular relaxation when linked to the locally produced nucleotide. However, in some vessels, the presence of P2Y in the smooth muscle can cause direct relaxation when it binds ATP released in a neural manner by the connection with purinergic or sensory nerves [106, 107]. Thus, P2 assumes the role of an attenuator of inflammation, vasodilating and protecting central organ [81, 100–103] but also inducing heart injury, while P1 acts as a cardioprotector [105].

Therefore, the role of P2 is uncertain, although P1 is active in protecting the heart. Thus, in the condition of hypoxia and ischaemia-reperfusion, Ado receptors are anti-inflammatory [108], as is the inhibition of P2 receptors, especially P2X7, because the expression of this subtype is increased in this situation, and they have therapeutic potential in critical infections with SARS-CoV-2 [91].

The importance of Ado A1 receptor ischaemic preconditioning in the heart should be considered in the context of COVID-19. Ado is involved in classic preconditioning and acts in particular through adenosine A1 and A3 receptors. A recent study demonstrated that remote ischaemic preconditioning activates adenosine A1 receptors during early reperfusion which induced Akt/endothelial nitric oxide (NO) synthase phosphorylation and improved mitochondrial function, thereby reducing the myocardial infarct size [109].

### 6.2. Purinergic Signalling in Heart Failure and Acute Coronary Syndrome

The development of heart failure causes structural cardiac damage to the point of leading to increased leukocyte infiltration, such that, in a situation of generalized inflammation, the purinergic system can act on the immune system through the action of CD73 acting in an anti-inflammatory way [110]. Studies provided evidence of the anti-inflammatory role of CD73 in T cells in the context of heart failure induced by transverse aortic constriction (TAC), probably related to antifibrotic activity and reduced production of proinflammatory cytokines by activating the A2A Ado receptor [110]. Ado therapy therefore has a cardioprotective role, however, with the signalling of A1R and A3R [111–114], while other studies used P2XR as a potent mediator [115]. However, the commitment of Ado action was associated with worsening CHF pathophysiology in another study [116].

In inflammatory conditions such as those associated with COVID-19, cell injury, and cardiac dysfunction with pulmonary lipopolysaccharide, A2AR deletion increased the inflammatory levels according to studies of interleukin levels, systemic inflammatory stress (haptoglobin and C-reactive protein), and myocardial injury by increased troponin I [117].

In the case of ACS, other investigators used P2Y12 inhibitors to reduce complications, including antithrombotic regimens that included apixaban, without aspirin, resulting in less bleeding and fewer hospitalizations without significant differences in the incidence of ischaemic events [117]. Similarly, ticagrelor was recognized as a new oral antagonist of the P2Y12 adenosine diphosphate receptor, with faster onset and with more significant platelet inhibition function in patients with ACS [118].

## 7. Conclusion

The main mechanisms of cardiovascular diseases caused by SARS-CoV-2 infection are related to host immune responses to viral invasion. Thus, the three mechanisms elucidated in this review result in myocardial damage, inflammatory processes, extensive release of proinflammatory cytokines and chemokines, and activation of immune cells. This exacerbation of the immune system generates CS that is responsible for increased levels of IL-2, IL-6, IL-7, IL-10, TNF-*α*, granulocyte colony-stimulating factor (G-CSF), IP-10, MCP-1, and macrophage inflammatory protein 1-alpha (MIP-1*α*), suggesting a systemic inflammatory process in critically ill patients.

COVID-19 infection has been associated with myocardial lesions in critical cases. Bearing in mind that the main damage pathways include exacerbations of inflammatory responses, the purinergic system has a high therapeutic potential in patients with cardiovascular diseases affected by COVID-19 in order to reduce the damage or prevent the progress of the disease by modulating immune responses. The development of anti-inflammatory therapies with P1R might reduce inflammatory levels in the heart, especially therapies targeting A2A, A1, and A3 in heart failure and heart dysfunction. In addition, the P2Y2 subtype should be studied in myocardial ischaemia, whereas, in cases of hypoxia and reperfusion-ischaemia, P2X7 inhibition appears effective.

Therefore, considering the immunomodulatory potential of the purinergic system, we believe that the blockade of P2X1R, P2X4R P2X7R, P2Y1R, and P2Y2R and upregulation of A2AR and A3R could be ideal mechanisms for pharmacological therapy of myocardial injury caused by CS in COVID-19. In addition, the suppression of extracellular ATP may be a strategy to inhibit purinergic signalling at P2 receptors, while increased Ado levels appear to reduce the immune response. These interventions may reduce systemic inflammatory damage to cells and tissues, preventing disease progression and modulating the immune response.

## Figures and Tables

**Table 1:** Cardiological findings related to disease severity and mortality.

Exam	Result	Association	Reference
NT-proBNP	High level	Heart failure	[28, 29]
Troponin (I or T)	High sensitivity	Myocardial injury	[28, 30]
D-dimer	Greater than 2.0 *μ*g/mL	Thrombosis	[28, 31, 34]
Prothrombin time	Longer	Investigation of coagulopathies	[28, 34]
Fibrinogen	High level	Analysis of consumption coagulopathies	[28, 34]
Full blood count	Lymphocytes and platelet count	Anaemia, leucocytosis/leucopenia, lymphopenia, thrombocytopenia	[28]
Procalcitonin	High level	Inflammatory marker, evaluation of bacterial coinfection	[28, 32]
C-reactive protein	High level	Inflammatory marker	[28, 32]
IL-6	High level	Inflammatory marker	[28, 32, 33]
Ferritin	Increases	Infection/inflammatory response	[28, 33]
Lactate dehydrogenase (LDH)	High level	Tissue damage	[35, 36]
Cardiac computed tomography (CT)	Used in uncertain cases with elevated troponins	Cardiac injury	[19]
ECG	Changes include ST and PR segment elevation and T and Q waves	Cardiac injury	[19]
Echocardiography	—	Myocardial systolic dysfunction and demonstrates myocyte necrosis and mononuclear cell infiltration	[19]

## References

[1] (2020). Coronavirus disease 2019. https://www.who.int/emergencies/diseases/novel-coronavirus-2019.

[2] Huang C., Wang Y., Li X. (2020). Clinical features of patients infected with 2019 novel coronavirus in Wuhan, China. *The Lancet*.

[3] Lai C. C., Shih T. P., Ko W. C., Tang H. J., Hsueh P. R. (2020). Severe acute respiratory syndrome coronavirus 2 (SARS-CoV-2) and coronavirus disease-2019 (COVID-19): the epidemic and the challenges. *International Journal of Antimicrobial Agents*.

[4] Liu P., Chen W., Chen J. P. (2019). Viral metagenomics revealed Sendai virus and coronavirus infection of Malayan pangolins (Manis javanica). *Viruses*.

[5] Ye Q., Wang B., Mao J. (2020). The pathogenesis and treatment of the ‘cytokine storm’ in COVID-19. *Journal of Infection*.

[6] Bagatini M. D., Bertolin K., Bridi A. (2019). 1*α*, 25-Dihydroxyvitamin D3 alters ectonucleotidase expression and activity in human cutaneous melanoma cells. *Journal of Cellular Biochemistry*.

[7] Wan S., Yi Q., Fan S. (2020). Relationships among lymphocyte subsets, cytokines, and the pulmonary inflammation index in coronavirus (COVID-19) infected patients. *British Journal of Haematology*.

[8] Tian S., Hu N., Lou J. (2020). Characteristics of COVID-19 infection in Beijing. *Journal of Infection*.

[9] Charlet R., Sendid B., Kaveri S. V., Poulain D., Bayry J., Jawhara S. (2019). Intravenous immunoglobulin therapy eliminates Candida albicans and maintains intestinal homeostasis in a murine model of dextran sulfate sodium-induced colitis. *International Journal of Molecular Sciences*.

[10] Zhou F., Yu T., Du R. (2020). Clinical course and risk factors for mortality of adult inpatients with COVID-19 in Wuhan, China: a retrospective cohort study. *The Lancet*.

[11] Wu Z., McGoogan J. M. (2020). Characteristics of and important lessons from the coronavirus disease 2019 (COVID-19) outbreak in China. *JAMA*.

[12] Zheng Z., Peng F., Xu B. (2020). Risk factors of critical & mortal COVID-19 cases: a systematic literature review and meta-analysis. *Journal of Infection*.

[13] Akhmerov A., Marbán E. (2020). COVID-19 and the heart. *Circulation Research*.

[14] Siddiqi H. K., Mehra M. R. (2020). COVID-19 illness in native and immunosuppressed states: a clinical–therapeutic staging proposal. *The Journal of Heart and Lung Transplantation*.

[15] Zhang W., Zhao Y., Zhang F. (2020). The use of anti-inflammatory drugs in the treatment of people with severe coronavirus disease 2019 (COVID-19): the perspectives of clinical immunologists from China. *Journal of Infection*.

[16] Wang D., Hu B., Hu C. (2020). Clinical characteristics of 138 hospitalized patients with 2019 novel coronavirus-infected pneumonia in Wuhan, China. *JAMA*.

[17] Kindler E., Thiel V. (2016). SARS-CoV and IFN: too little, too late. *Cell Host & Microbe*.

[18] De Wit E., Van Doremalen N., Falzarano D., Munster V. J. (2016). SARS and MERS: recent insights into emerging coronaviruses. *Nature Reviews Microbiology*.

[19] Prompetchara E., Ketloy C., Palaga T. (2020). Immune responses in COVID-19 and potential vaccines: lessons learned from SARS and MERS epidemic. *Asian Pacific Journal of Allergy and Immunology*.

[20] Channappanavar R., Fehr A. R., Vijay R. (2016). Dysregulated type I interferon and inflammatory monocyte-macrophage responses cause lethal pneumonia in SARS-CoV-infected mice. *Cell Host & Microbe*.

[21] di Mauro G., Scavone C., Rafaniello C., Rossi F., Capuano A. (2020). SARS-CoV-2 infection: response of human immune system and possible implications for the rapid test and treatment. *International Immunopharmacology*.

[22] Tu Y. F., Chien C. S., Yarmishyn A. A. (2020). A review of SARS-CoV-2 and the ongoing clinical trials. *International Journal of Molecular Sciences*.

[23] Guzik T. J., Mohiddin S. A., Dimarco A. (2020). COVID-19 and the cardiovascular system: implications for risk assessment, diagnosis, and treatment options. *Cardiovascular Research*.

[24] Kochi A. N., Tagliari A. P., Forleo G. B., Fassini G. M., Tondo C. (2020). Cardiac and arrhythmic complications in patients with COVID-19. *Journal of Cardiovascular Electrophysiology*.

[25] Xiong T. Y., Redwood S., Prendergast B., Chen M. (2020). Coronaviruses and the cardiovascular system: acute and long-term implications. *European Heart Journal*.

[26] Babapoor-Farrokhran S., Gill D., Walker J., Rasekhi R. T., Bozorgnia B., Amanullah A. (2020). Myocardial injury and COVID-19: possible mechanisms. *Life Sciences*.

[27] Zheng Y. Y., Ma Y. T., Zhang J. Y., Xie X. (2020). COVID-19 and the cardiovascular system. *Nature Reviews Cardiology*.

[28] Moccia F., Gerbino A., Lionetti V. (2020). COVID-19-associated cardiovascular morbidity in older adults: a position paper from the Italian Society of Cardiovascular Researches. *GeroScience*.

[29] Lau S.-T., Yu W.-C., Mok N.-S., Tsui P.-T., Tong W.-L., Cheng S. W. C. (2005). Tachycardia amongst subjects recovering from severe acute respiratory syndrome (SARS). *International Journal of Cardiology*.

[30] Badawi A., Ryoo S. G. (2016). Prevalence of comorbidities in the Middle East respiratory syndrome coronavirus (MERS-CoV): a systematic review and meta-analysis. *International Journal of Infectious Diseases*.

[31] Madjid M., Connolly A. T., Nabutovsky Y., Safavi-Naeini P., Razavi M., Miller C. C. (2019). Effect of high influenza activity on risk of ventricular arrhythmias requiring therapy in patients with implantable cardiac defibrillators and cardiac resynchronization therapy defibrillators. *The American Journal of Cardiology*.

[32] Favaloro E. J., Lippi G. (2020). Recommendations for minimal laboratory testing panels in patients with COVID-19: potential for prognostic monitoring. *Seminars in Thrombosis and Hemostasis*.

[33] Guo T., Fan Y., Chen M. (2020). Cardiovascular implications of fatal outcomes of patients with coronavirus disease 2019 (COVID-19). *JAMA Cardiology*.

[34] Ohtsuki I., Morimoto S. (2013). Troponin. *Encyclopedia of Biological Chemistry: Second Edition*.

[35] Zhang L., Yan X., Fan Q. (2020). D-dimer levels on admission to predict in-hospital mortality in patients with COVID-19. *Journal of Thrombosis and Haemostasis*.

[36] Liu F., Li L., Xu M. (2020). Prognostic value of interleukin-6, C-reactive protein, and procalcitonin in patients with COVID-19. *Journal of Clinical Virology*.

[37] Henry B. M., De Oliveira M. H. S., Benoit S., Plebani M., Lippi G. (2020). Hematologic, biochemical and immune biomarker abnormalities associated with severe illness and mortality in coronavirus disease 2019 (COVID-19): a meta-analysis. *Clinical Chemistry and Laboratory Medicine*.

[38] Tang N., Li D., Wang X., Sun Z. (2020). Abnormal coagulation parameters are associated with poor prognosis in patients with novel coronavirus pneumonia. *Journal of Thrombosis and Haemostasis*.

[39] Shi S., Qin M., Shen B. (2020). Association of cardiac injury with mortality in hospitalized patients with COVID-19 in Wuhan, China. *JAMA Cardiology*.

[40] Zeng J. H., Liu Y. X., Yuan J. (2020). First case of COVID-19 complicated with fulminant myocarditis: a case report and insights. *Infection*.

[41] Bansal M. (2020). Cardiovascular disease and COVID-19. *Diabetes & Metabolic Syndrome: Clinical Research & Reviews*.

[42] Fan G., Yu J., Asare P. F. (2016). Danshensu alleviates cardiac ischaemia/reperfusion injury by inhibiting autophagy and apoptosis via activation of mTOR signalling. *Journal of Cellular and Molecular Medicine*.

[43] Lu R., Zhao X., Li J. (2020). Genomic characterisation and epidemiology of 2019 novel coronavirus: implications for virus origins and receptor binding. *The Lancet*.

[44] Sungnak W., Huang N., Bécavin C. (2020). SARS-CoV-2 entry factors are highly expressed in nasal epithelial cells together with innate immune genes. *Nature Medicine*.

[45] Shulla A., Heald-Sargent T., Subramanya G., Zhao J., Perlman S., Gallagher T. (2011). A transmembrane serine protease is linked to the severe acute respiratory syndrome coronavirus receptor and activates virus entry. *Journal of Virology*.

[46] Jawhara S. (2020). Could intravenous immunoglobulin collected from recovered coronavirus patients protect against COVID-19 and strengthen the immune system of new patients?. *International Journal of Molecular Sciences*.

[47] Monto A. S., Cowling B. J., Peiris J. S. M. (2014). Coronaviruses. *Viral Infections of Humans: Epidemiology and Control*.

[48] Clerkin K. J., Fried J. A., Raikhelkar J. (2020). COVID-19 and cardiovascular disease. *Circulation*.

[49] Pinto B. G. G., Oliveira A. E. R., Singh Y. (2020). ACE2 expression is increased in the lungs of patients with comorbidities associated with severe COVID-19. *The Journal of Infectious Diseases*.

[50] Cheng H., Wang Y., Wang G. Q. (2020). Organ-protective effect of angiotensin-converting enzyme 2 and its effect on the prognosis of COVID-19. *Journal of Medical Virology*.

[51] Rizzo P. (2020). COVID-19 in the heart and the lungs: could we “Notch” the inflammatory storm?. *Basic Research in Cardiology*.

[52] Cavezzi A., Troiani E., Corrao S. (2020). COVID-19: hemoglobin, iron, and hypoxia beyond inflammation. A narrative review. *Clinics and Practice*.

[53] Yang X., Fu J., Wan H. (2019). Protective roles and mechanisms of taurine on myocardial hypoxia/reoxygenation-induced apoptosis. *Acta Cardiologica Sinica*.

[54] Zhao M., Wang M., Zhang J. (2020). Advances in the relationship between coronavirus infection and cardiovascular diseases. *Biomedicine & Pharmacotherapy*.

[55] Oldstone M. B. A., Rosen H. (2014). Sphingosine-1-phosphate signaling in immunology and infectious diseases. *Current Topics in Microbiology and Immunology*.

[56] Mehta P., McAuley D. F., Brown M., Sanchez E., Tattersall R. S., Manson J. J. (2020). COVID-19: consider cytokine storm syndromes and immunosuppression. *The Lancet*.

[57] Cheung C. Y., Poon L. L. M., Ng I. H. Y. (2005). Cytokine responses in severe acute respiratory syndrome coronavirus-infected macrophages in vitro: possible relevance to pathogenesis. *Journal of Virology*.

[58] McGonagle D., Sharif K., O’Regan A., Bridgewood C. (2020). The role of cytokines including interleukin-6 in COVID-19 induced pneumonia and macrophage activation syndrome-like disease. *Autoimmunity Reviews*.

[59] Diao B., Wang C., Tan Y. (2020). Reduction and functional exhaustion of T cells in patients with coronavirus disease 2019 (COVID-19). *Frontiers in Immunology*.

[60] Henderson L. A., Canna S. W., Schulert G. S. (2020). On the alert for cytokine storm: immunopathology in COVID-19. *Arthritis & Rhematology*.

[61] Zhang C., Wu Z., Li J. W., Zhao H., Wang G. Q. (2020). Cytokine release syndrome in severe COVID-19: interleukin-6 receptor antagonist tocilizumab may be the key to reduce mortality. *International Journal of Antimicrobial Agents*.

[62] Yang Y., Shen C., Li J. (2020). Exuberant elevation of IP-10, MCP-3 and IL-1ra during SARS-CoV-2 infection is associated with disease severity and fatal outcome.

[63] Zhang J., Xie B., Hashimoto K. (2020). Current status of potential therapeutic candidates for the COVID-19 crisis. *Brain, Behavior, and Immunity*.

[64] Janka G. E., Lehmberg K. (2014). Hemophagocytic syndromes - an update. *Blood Reviews*.

[65] Zhu H., Rhee J.-W., Cheng P. (2020). Cardiovascular complications in patients with COVID-19: consequences of viral toxicities and host immune response. *Current Cardiology Reports*.

[66] Chen N., Zhou M., Dong X. (2020). Epidemiological and clinical characteristics of 99 cases of 2019 novel coronavirus pneumonia in Wuhan, China: a descriptive study. *The Lancet*.

[67] Siripanthong B., Nazarian S., Muser D. (2020). Recognizing COVID-19-related myocarditis: the possible pathophysiology and proposed guideline for diagnosis and management. *Heart Rhythm*.

[68] Inciardi R. M., Lupi L., Zaccone G. (2020). Cardiac involvement in a patient with coronavirus disease 2019 (COVID-19). *JAMA Cardiology*.

[69] Friedrich M. G., Sechtem U., Schulz-Menger J. (2009). Cardiovascular magnetic resonance in myocarditis: a JACC White Paper. *Journal of the American College of Cardiology*.

[70] Driggin E., Madhavan M. V., Bikdeli B. (2020). Cardiovascular considerations for patients, health care workers, and health systems during the COVID-19 pandemic. *Journal of the American College of Cardiology*.

[71] Van Linthout S., Tschöpe C. (2018). Viral myocarditis: a prime example for endomyocardial biopsy-guided diagnosis and therapy. *Current Opinion in Cardiology*.

[72] Hendren N. S., Drazner M. H., Bozkurt B., Cooper L. T. (2020). Description and proposed management of the acute COVID-19 cardiovascular syndrome. *Circulation*.

[73] Kang Y., Chen T., Mui D. (2020). Cardiovascular manifestations and treatment considerations in COVID-19. *Heart*.

[74] Khan I. H., Zahra S. A., Zaim S., Harky A. (2020). At the heart of COVID-19. *Journal of Cardiac Surgery*.

[75] Tang N., Bai H., Chen X., Gong J., Li D., Sun Z. (2020). Anticoagulant treatment is associated with decreased mortality in severe coronavirus disease 2019 patients with coagulopathy. *Journal of Thrombosis and Haemostasis*.

[76] Iba T., Levy J. H., Levi M., Connors J. M., Thachil J. (2020). Coagulopathy of coronavirus disease 2019. *Critical Care Medicine*.

[77] Ullah W., Saeed R., Sarwar U., Patel R., Fischman D. L. (2020). COVID-19 complicated by acute pulmonary embolism and right-sided heart failure. *JACC: Case Reports*.

[78] Vivas D., Roldán V., Esteve-Pastor M. A. (2020). Recommendations on antithrombotic treatment during the COVID-19 pandemic. Position statement of the Working Group on Cardiovascular Thrombosis of the Spanish Society of Cardiology. *Revista Española de Cardiología*.

[79] Burnstock G., Pelegrín P. (2020). Introduction to purinergic signaling. *Purinergic Signaling. Methods in Molecular Biology*.

[80] Cronstein B. N., Sitkovsky M. (2017). Adenosine and adenosine receptors in the pathogenesis and treatment of rheumatic diseases. *Nature Reviews Rheumatology*.

[81] Eltzschig H. K., Sitkovsky M. V., Robson S. C. (2012). Purinergic signaling during inflammation. *New England Journal of Medicine*.

[82] Sitkovsky M. V. (2003). Use of the A 2A adenosine receptor as a physiological immunosuppressor and to engineer inflammation in vivo. *Biochemical Pharmacology*.

[83] Hiley C. R., Bottrill F. E., Wamock J., Richardson P. J. (1995). Effects of pH on responses to adenosine, CGS 21680, carbachol and nitroprusside in the isolated perfused superior mesenteric arterial bed of the rat. *British Journal of Pharmacology*.

[84] Burnstock G., Dale N. (2015). Purinergic signalling during development and ageing. *Purinergic Signal*.

[85] Burnstock G. (2018). The therapeutic potential of purinergic signalling. *Biochemical Pharmacology*.

[86] Li H., Liu L., Zhang D. (2020). SARS-CoV-2 and viral sepsis: observations and hypotheses. *The Lancet*.

[87] Di Virgilio F., Tang Y., Sarti A. C., Rossato M. (2020). A rationale for targeting the P2X7 receptor in coronavirus disease 19. *British Journal of Pharmacology*.

[88] Burnstock G., Knight G. E. (2018). The potential of P2X7 receptors as a therapeutic target, including inflammation and tumour progression. *Purinergic Signal*.

[89] Mehta N., Kaur M., Singh M. (2014). Purinergic receptor P2X7: a novel target for anti-inflammatory therapy. *Bioorganic & Medicinal Chemistry*.

[90] Di Virgilio F., Dal Ben D., Sarti A. C., Giuliani A. L., Falzoni S. (2017). The P2X7 receptor in infection and inflammation. *Immunity*.

[91] Burnstock G. (2016). P2X ion channel receptors and inflammation. *Purinergic Signal*.

[92] Ruiz-Rodríguez V. M., Cortes-García J. D., de Jesús B.-E. M. (2019). P2X4 receptor as a modulator in the function of P2X receptor in CD4+ T cells from peripheral blood and adipose tissue. *Molecular Immunology*.

[93] Suurväli J., Boudinot P., Kanellopoulos J., Boudinot S. R. (2020). P2X4: a fast and sensitive purinergic receptor. *Biomedical Journal*.

[94] Antonioli L., Blandizzi C., Fornai M., Pacher P., Lee H. T., Haskó G. (2019). P2X4 receptors, immunity, and sepsis. *Current Opinion in Pharmacology*.

[95] Paalme V., Rump A., Mädo K. (2019). Human peripheral blood eosinophils express high levels of the purinergic receptor P2X4. *Frontiers in Immunology*.

[96] Guerra A. N., Fisette P. L., Pfeiffer Z. A. (2003). Purinergic receptor regulation of LPS-induced signaling and pathophysiology. *Journal of Endotoxin Research*.

[97] Lazarowski E. R., Boucher R. C. (2001). UTP as an extracellular signaling molecule. *News in Physiological Sciences*.

[98] Cauwels A., Rogge E., Vandendriessche B., Shiva S., Brouckaert P. (2014). Extracellular ATP drives systemic inflammation, tissue damage and mortality. *Cell Death & Disease*.

[99] Granado M., Amor S., Montoya J. J., Monge L., Fernández N., García-Villalón Á. L. (2015). Altered expression of P2Y2 and P2X7 purinergic receptors in the isolated rat heart mediates ischemia-reperfusion injury. *Vascular Pharmacology*.

[100] Thompson L. F., Eltzschig H. K., Ibla J. C. (2004). Crucial role for ecto-5′-nucleotidase (CD73) in vascular leakage during hypoxia. *The Journal of Experimental Medicine*.

[101] Grenz A., Zhang H., Eckle T. (2007). Protective role of ecto-5′-nucleotidase (CD73) in renal ischemia. *Journal of the American Society of Nephrology*.

[102] Eckle T., Füllbier L., Wehrmann M. (2007). Identification of ectonucleotidases CD39 and CD73 in innate protection during acute lung injury. *Journal of Immunology*.

[103] Hart M. L., Grenz A., Gorzolla I. C., Schittenhelm J., Dalton J. H., Eltzschig H. K. (2011). Hypoxia-inducible factor-1*α*–dependent protection from intestinal ischemia/reperfusion Injury involves ecto-5′-nucleotidase (CD73) and the A2B adenosine receptor. *Journal of Immunology*.

[104] Xiang F., Huang Y. S., Zhang D. X., Chu Z. G., Zhang J. P., Zhang Q. (2010). Adenosine A1 receptor activation reduces opening of mitochondrial permeability transition pores in hypoxic cardiomyocytes. *Clinical and Experimental Pharmacology & Physiology*.

[105] Burnstock G., Pelleg A. (2015). Cardiac purinergic signalling in health and disease. *Purinergic Signal*.

[106] Burnstock G., Kennedy C. (1986). A dual function for adenosine 5′-triphosphate in the regulation of vascular tone. *Circulation Research*.

[107] Ralevic V., Burnstock G. (1991). Roles of P2-purinoceptors in the cardiovascular system. *Circulation*.

[108] Salvatore C. A., Tilley S. L., Latour A. M., Fletcher D. S., Koller B. H., Jacobson M. A. (2000). Disruption of the A3 adenosine receptor gene in mice and its effect on stimulated inflammatory cells. *The Journal of Biological Chemistry*.

[109] Paez D. T., Garces M., Calabró V. (2019). Adenosine A1receptors and mitochondria: targets of remote ischemic preconditioning. *American Journal of Physiology-Heart and Circulatory Physiology*.

[110] Quast C., Alter C., Ding Z., Borg N., Schrader J. (2017). Adenosine formed by CD73 on T cells inhibits cardiac inflammation and fibrosis and preserves contractile function in transverse aortic constriction-induced heart failure. *Circulation: Heart Failure*.

[111] Kitakaze M., Minamino T., Node K. (1999). Adenosine and cardioprotection in the diseased heart. *Circulation Journal*.

[112] Jang Eun L., Gary B., Bruce T. L. (2001). A novel cardioprotective role of RhoA: new signaling mechanism for adenosine. *The FASEB Journal*.

[113] Liang B. T., Jacobson K. A. (1998). A physiological role of the adenosine A3 receptor: sustained cardioprotection. *Proceedings of the National Academy of Sciences of the United States of America*.

[114] Dougherty C., Barucha J., Schofield P. R., Jacobson K. A., Liang B. T. (1998). Cardiac myocytes rendered ischemia resistant by expressing the human adenosine A 1 or A 3 receptor. *The FASEB Journal*.

[115] Santhosh Kumar T., Zhou S. Y., Joshi B. V. (2010). Structure-activity relationship of (N)-methanocarba phosphonate analogues of 5′-AMP as cardioprotective agents acting through a cardiac P2X receptor. *Journal of Medicinal Chemistry*.

[116] Asakura M., Asanuma H., Kim J. (2007). Impact of adenosine receptor signaling and metabolism on pathophysiology in patients with chronic heart failure. *Hypertension Research*.

[117] Lopes R. D., Heizer G., Aronson R. (2019). Antithrombotic therapy after acute coronary syndrome or PCI in atrial fibrillation. *The New England Journal of Medicine*.

[118] Wang D., Yang X. H., Zhang J. D., Li R.-B., Jia M., Cui X. R. (2018). Compared efficacy of clopidogrel and ticagrelor in treating acute coronary syndrome: a meta-analysis. *BMC Cardiovascular Disorders*.

